# Biosynthesis of Silver Nanoparticles and Their Applications in Harvesting Sunlight for Solar Thermal Generation

**DOI:** 10.3390/nano11092421

**Published:** 2021-09-17

**Authors:** Rebwar Faiq Talabani, Samir Mustafa Hamad, Azeez Abdullah Barzinjy, Usame Demir

**Affiliations:** 1 Department of Mechanical Engineering, Engineering and Architecture Faculty, Bingöl University, 12000 Bingöl, Turkey; r_talabany@yahoo.com (R.F.T.); udemir@bingol.edu.tr (U.D.); 2 Scientific Research Centre, Soran University, Soran 44008, Iraq; samir.hamad@soran.edu.iq; 3 Department of Physics, College of Education, Salahaddin University-Erbil, Erbil 44002, Iraq; 4 Department of Physics Education, Faculty of Education, Tishk International University, Erbil 44001, Iraq

**Keywords:** silver nanofluid, green synthesis method, surface plasmon resonance effect, photothermic energy, parsley

## Abstract

Silver (Ag) nanoparticles (NPs) have been synthesized through an easy, inexpensive, and ecofriendly method. *Petroselinum crispum*, parsley, leaf extract was utilized as a reducing, capping, and stabilizing agent, without using any hazardous chemical materials, for producing Ag NPs. The biosynthesized Ag NPs were characterized using different characterization techniques, namely UV-Vis, FT-IR spectroscopy, X-ray diffraction (XRD), X-ray Photoelectron Spectroscopy (XPS), dynamic light scattering (DLS), zeta potential, differential scanning calorimetry (DSC), thermogravimetric analysis (TGA), transmission electron microscope (TEM), field emission scanning electron microscopy (FESEM), and energy-dispersive X-ray (EDX) analysis to investigate the optical, thermal, structural, morphological, and chemical properties of the plant extract and the biosynthesized Ag NPs. After that, the biosynthesized Ag NPs were utilized in harvesting sunlight for solar thermal generation. Surface plasmon resonance (SPR) for the green synthesized Ag NPs with the dark color were adjusted at nearly 450 nm. Once the Ag NPs are excited at the SPR, a large amount of heat is released, which causes a change in the local refractive index surrounding the Ag NPs. The released heat from the Ag NPs under the solar irradiation at the precise wavelength of plasmon resonance significantly increased the temperature of the aqueous medium. Different percentages of Ag NPs were dispersed in water and then exposed to the sunlight to monitor the temperature of the suspension. It was found that the temperature of the aqueous medium reached its highest point when 0.3 wt. % of Ag NPs was utilized. This investigation is rare and unique, and it shows that utilizing a small amount of the biosynthesized Ag NPs can increase the temperature of the aqueous medium remarkably.

## 1. Introduction

The demographic and economic growth of our modern society has led to high demand for energy, which is largely met by the use of fossil fuels. [1]. However, because of its limited availability and the negative impact on the environment, it is important to build technologies that allow for more effective use of alternative energy sources [2]. Solar energy, in general, is safe, abundant, simple to obtain, and of unlimited supply. Accordingly, solar radiation energy is one of the most promising sources for meeting future energy demand [3]. Similarly, solar energy can be effectively transformed into electrical and thermal energy through the photovoltaic and photocatalytic processes [4]. Therefore, metal-based nanomaterials are the most common photoactive materials capable of performing the processes described above [5]. In turn, nanoparticles (NPs), which are particles with one or more dimensions within the range of 100 nanometers or less, have attracted great interest due to their unique and attractive features and their irreplaceable usage over their analogues bulky materials [6]. Metallic nanoparticles, in general, possess numerous advantages. First, their optical properties can be easily controlled; practically, they are perfect optical absorbers [7]. Second, they can be utilized in multifunctional applications to accomplish some specific purposes such as rapid thermal response [8], corrosion resistance [9], recyclability [10], etc. Last but not least, through the doping of metal-based materials, heat loss can be effectively reduced [11]. 

Photoactive nanomaterials, composed of novel metals, are capable of converting solar energy into thermal energy [12]. In recent times, the synthesis of metallic NPs is an important area of research due to their diverse application [13]. When the particle size goes to the nanoscale, its catalytic [14], optical [7], thermal [15,16], mechanical [17], electronic [18], and magnetic activity [19] improve significantly. Accordingly, metallic NPs can be used in different areas of application, such as absorption of light spectrum [20], water purification [21], antimicrobial activity [13], biomedicine [22], sensors [23], and many others. 

Among the metallic NPs, silver (Ag) NPs show great potential in the scientific community due to their wide range of applications [24]. In fact, there are three main methods for synthesizing nanomaterials, namely physical, chemical, and biological or green methods [25,26]. The physical methods required highly sophisticated instruments, high pressure, and high temperature [27], while the chemical methods have a hazardous impact on the producers and users due to the utilizing precarious chemical materials as reducing, capping, and stabilizing agents [28]. Thus, they cause many difficulties for the human and the environment at the same time. Accordingly, in recent times, researchers have focused on the biological or green method for synthesizing nanomaterials [29]. In addition, the fabrication of nanomaterials using biological method is gaining more advantage due to its simplicity, eco-friendliness, low cost, and toxic chemicals avoidance [30]. This great advantage of non-toxic synthesis facilitates the possibility of using these nanomaterials, especially in products that are closely related to humans, such as shampoo, toothpaste, and photothermic conversion therapy.

Green synthesis of NPs includes using natural materials such as plants and microorganisms, e.g., bacteria, fungi, algae, and yeasts [30]. However, the existing phytochemicals in plant extracts possess an exceedingly high ability for reducing metal ions within a short time as compared with bacteria, fungi, algae, and yeasts, which necessitates a longer incubation period [31,32]. As a result, plant extracts have been noted as a prominent source for the synthesis of metallic NPs. In addition, the plant-mediated synthesis procedure for synthesizing NPs is a leading process over the microorganism process due to its simplicity, rapidity, and avoidance of culture maintenance [33]. Furthermore, plant supplies, such as flowers, leaves, seeds, stems, fruits, and peels, have been used as reducing and capping agents in the NPs fabrication process [34]. Generally, plant extractions possess a significant prominence due to involving a large number of phytochemicals such as flavonoids, glycosides, polyphenol, terpenoids, and enzymes, which act as reducing, capping, and stabilizing agents [35]. Additionally, the functional groups, e.g., -C=C- and -C=O, present in phytochemicals can also contribute to the production of nanoparticles [36]. Moreover, green methods designate an environmentally friendly production of nanoparticles of different sizes and shapes [37,38].

This investigation is focusing on Ag NPs, due to the importance of Ag NPs whose optical properties depend on their size and shape. This dependency, most likely, arises from the surface plasmon resonance (SPR) and free electrons of the nanomaterials [39]. In fact, the plasmonic effect describes the interactions of light with metallic nanoparticles [40]. Furthermore, SPR is the resonant oscillation of the free electrons at the metal surface layer, which are excited by incident light sources. Resonant frequencies for noble metals, such as Ag, are located in the middle of the optical spectrum; thus, SPR for such metals interact strongly with light and depend on the size and shape of the NPs [41,42]. Plasmonic is a part of nanotechnology where nanostructures are used as an active element to focus, direct, control, and manipulate light. Even though the interaction of light and metallic NPs has long concerned the interest of scientists, plasmonic signifies a rather new level of control and study, including both nanostructures and light [43]. Moreover, the plasmonic effect in metallic NPs is a multidisciplinary and important area of research owing to its potential applications in optoelectronic devices [44], thermodynamic [45], sensors [46], and medical diagnosis [47].

In continuation of our recent works [48,49,50,51,52,53,54,55,56] regarding green synthesis nanomaterials, here in this study, an active and easy process of one-pot green synthesis of Ag NPs is proposed. The novelty of this research is that Ag NPs can be formed from a one-pot reaction deprived of employing any exterior stabilizing and reducing agent, which is not conceivable by means of the existing processes. Ag NPs were synthesized using *Petroselinum crispum* extract, commonly known as parsley. Parsley is belonging to the Apiaceae carrot family, and its chemical structure contains flavonoids, polyphenols, carotenoids, lipids, polysaccharides, tannins, and essential oils, which are considered bio-reducing and stabilizing agents. Polyphenols and flavonoids are the dominant compounds in the parsley extract [57,58]. Both parsley extract and Ag NPs have been characterized using different characterization techniques. Then, the biosynthesized Ag NPs from parsley extract were dissolved in water, and formerly their ability to perform the conversion of solar energy into heat energy, based on the plasmonic effect, was investigated for boiling water application.

## 2. Materials and Methods

### 2.1. Preparation of the Parsley Extract

Parsley was collected from Rashken (Latitude 36°11′58.0″ N and Longitude 43°56′54.7″ E) in Erbil city, Iraqi Kurdistan Region in the spring season (March 2021). Five grams of fresh parsley was soaked in a flask contained 100 mL double distilled water (DD water). The solution was heated at 80 °C for 40 min. The extract was allowed to cool down to room temperature and then filtered with a filter paper to remove unwanted organic materials. Subsequently, the pure filtrate extract was stored in the refrigerator for further experimental work.

### 2.2. Synthesis of Silver Nanoparticles

Silver nitrate, Ag NO_3_, molecular weight 169.87 g/mol and purity > 99%, was purchased from Sigma Aldrich company (Istanbul, Turkey) and used as received with unpolluted specialized status. The amount of 2 mg of silver nitrite was dissolved in 50 mL double distilled water and kept stirring for 20 min at 80 °C. Then, 50 mL of parsley extract solution was drop-wise added to the dissolved silver nitrite. The final mixture was put on the hotplate, heated, and stirred at 70 °C for 30 min until the color of the mixture changed to a brownish color. The new chromatic appearance of the mixture is considered a priority indicator for synthesizing Ag NPs. The obtained precipitates were separated from the mixture by centrifugation at 7000 rpm for 25 min and afterwards heated at 500 °C for 40 min using an oven to remove all of the impurities and organic materials around the Ag NPs.

**Figure 1:**
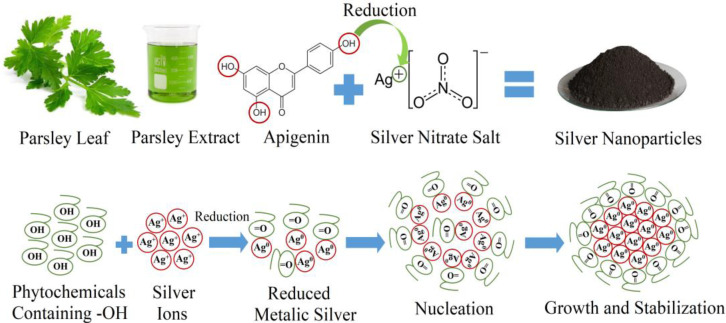
Proposed mechanism of biosynthesizing Ag NPs from parsley extract.

The mechanism of biosynthesizing Ag NPs can be explained through the following steps: first, the activation step which involves the reduction of the available metal ions into metal atoms; second, the nucleation and growth step, which includes the combination of the available atoms to form NPs of a conclusive size and shape; third, the stabilization step, in which the phytochemicals cap the NPs, therefore preventing them from agglomeration; and finally, calcination of NPs should be done to acquire pure NPs. Figure 1 represents the schematic diagram and the mechanism of Ag NPs formation by parsley extract. The agglomeration occurred due to the sturdier binding energy between two metal atoms; the agglomeration of NPs is prevented to some extent by the secondary metabolites of plants, which act as a capping and stabilizing agent.

### 2.3. Characterization of Ag NPs

X-ray diffraction (XRD) measurements were carried out using a PAN analytical X′Pert PRO (Cu Kα = 1.5406 A°). The scanning rate was 1°/min in the 2θ range from 20 to 80°. XRD can be used for the determination of crystal structure, purity, and crystallite size of the nanoparticles. X-ray Photoelectron Spectroscopy (XPS) spectra of Ag NPs were studied by means of a hemispherical analyzer (Physical Electronics 1257 system) (Partow Rayan company, Tehran, Iran). For the XPS, an identical anode (Mg and Al) with an X-ray basis was run at 400 W of persistent power with Al Kα radiation (1486.6 eV). The sample was located in a sample stage with a release angle of 45°. The analysis was supported by hanging Ag NPs on a gold film, whereas gold functioned as a metallic reference. Au 4f binding-energy was 84 eV for samples deprived of any charging effect. Furthermore, UV-Vis spectroscopy analysis was conducted using a double-beam spectrophotometer (Super Aquarius spectrophotometer-1000) Soran University, Soran, Erbil, Iraq) to confirm the formation of Ag NPs. Additionally, the morphology and particle dispersion were investigated by field emission scanning electron microscopy (FE-SEM) (Quanta 450) Soran University, Soran, Erbil, Iraq). The chemical composition of the prepared nanostructures was studied using energy-dispersive X-ray spectroscopy (EDX) performed in the FE-SEM instrument. The shape and size of the Ag NPs were characterized by a high-resolution transmission electron microscope (HRTEM) (Partow Rayan company, Tehran, Iran) utilizing a Philips (USA) EM208 microscope functioning at an accelerating voltage of 90 kV. Fourier transform infrared (FTIR) spectroscopy with a resolution of 4 cm^−1^ was used to investigate the functional groups in the leaf extract and the NPs independently. The actual size of the biosynthesized Ag NPs was computed by dynamic light scattering (DLS). The particle size was computed utilizing a Malvern Zetasizer 3000HSA (Malvern, Worcs, UK) equipped with a 10-mW He–Ne laser (633 nm) and functioning at an angle of 90° and a temperature of 20 °C. T. Differential scanning colorimetry (DSC) type (TA Instruments, (Partow Rayan Company, Tehran, Iran) in the range 50–1000 °C was utilized for the DSC curve. Thermogravimetric anal sis (TGA) was performed using Perkin-Elmer-Pyris1 analyzer (Partow Rayan Company, Tehran, Iran).

### 2.4. Increasing Temperature during Irradiation of the System

**Figure 2:**
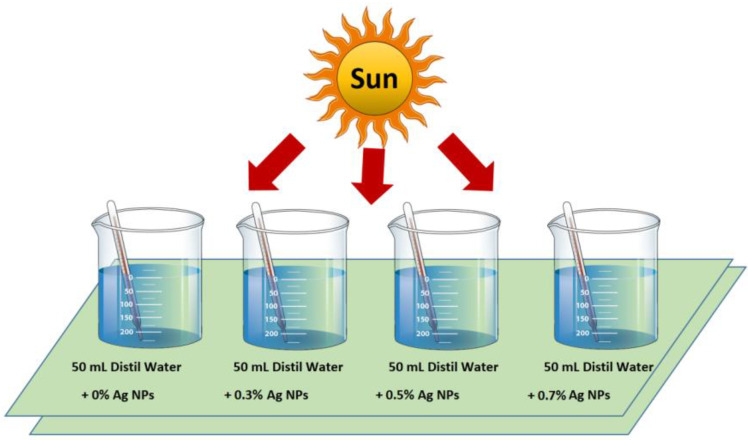
Representation diagram of rising temperature during sunlight irradiation using different concentrations of Ag NPs.

After the synthesis and characterization of Ag NPs by a green method using parsley extract, the NPs were utilized to increase the water temperature. A representation diagram of the present study is shown in Figure 2. The system contains four equalized bakers filled with 50 mL of distilled water.

Different concentrations of Ag NPs have been used in each beaker, and their initial temperature was recorded (14 °C) from their own fine thermometer (laboratory thermometer, Lafayette Township, New Jersey, USA). After that, the beakers were subjected to ultrasound to disperse the NPs homogeneously inside water and record the temperature of the solutions homogeneously. Then, the system was placed in front of the sunlight source to investigate the amount of the released heat by the utilized nanoparticles into the water medium. It should be noted that the temperature of the solution was not raised by thermal solar radiation, but rather the plasmonic effect was responsible for this in a way that the heat exchange between the water and the external conditions before the experiment was sufficient. The temperature of each beaker under the sunlight radiation was monitored sensibly, so there would be no temperature lag, and the values of temperature were registered every 2 min. The detail of Ag concentration and ambient temperatures are summarized in Table 1.

## 3. Results and Discussion

### 3.1. Characterization of Parsley Leaf Extract

**Figure 3:**
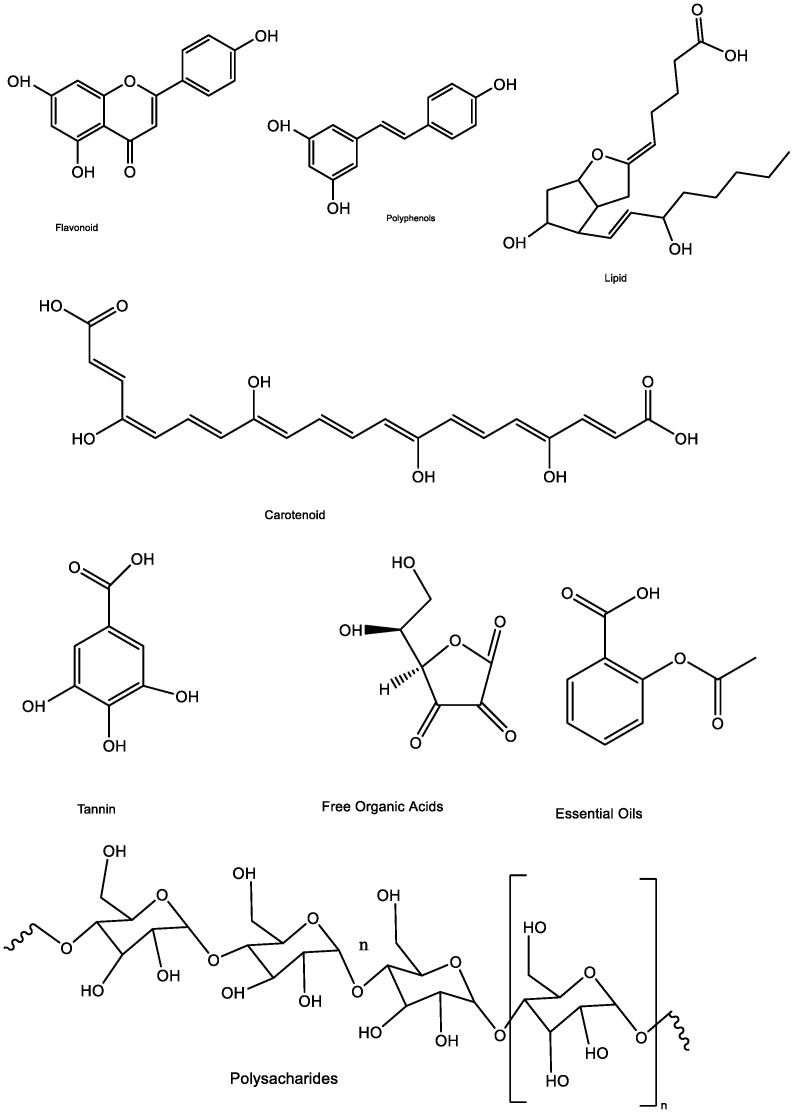
Available phytochemicals in Parsley leaf extract.

The available phytochemicals in parsley extract reduce the available metal ion in the silver nitrate salt to metal zero nanoparticles. Therefore, plant extract, at the same time, acts as a reducing and stabilizing agent. Makarov et al. [59] suggested that metal atoms would be compressed as organic casing in three steps for their degree of steadiness after reduction by plant extracts. Metal ion reduction and nucleation of the reduced metal atom would be in the activation phase, the NPs steadiness improved through the growth phase, and the shape of the NPs formed during the end phase. UV-Vis spectroscopy observes this reaction growth. The UV-Vis spectroscopy revealed an absorption peak, associated with the surface plasmon resonance (SPR), gathers conduction band electrons oscillations in responding with electromagnetic waves, demonstrating metal ion reduction and NPs formation [51]. Parsley leaf extract comprises flavonoids, polyphenols, carotenoids, lipids, polysaccharides, tannins, free organic acids, and essential oils (Figure 3), which are considered essential bio-reducing and stabilizing agents through possessing -OH groups for NPs formation [60,61]. Flavonoids and polyphenols are predominant compounds of Parsley [62]. These phytochemicals, being antioxidant and free from toxic chemicals, are tremendously able to reduce metal ions and stabilizing them in nanoscale length. These phytochemicals are also directly affecting the shape and size of the biosynthesized NPs [63].

**Figure 4:**
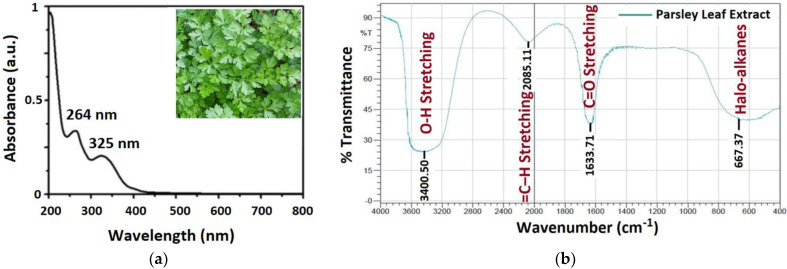
(**a**) UV-Vis spectra and (**b**) FTIR spectra of Parsley leaf extract.

Figure 4a displays the UV-Vis spectrum of Parsley leaf extract. We believed that the dominant peaks, at 264 nm and 325 nm, are more likely related to the phenolic components, i.e., polyphenols and flavonoid available in Parsley leaf extract. The functional organic molecules, for instance, Apiin, phenol, ascorbic acid, exist in the Parsley leaf extract [64]. Liu et al. identified that these peaks associated with Apiin, C_26_H_28_O_14_, a natural flavonoid, exist in Parsley leaf extract [65]. Markarov et al. [59] state that flavonoids, as a general rule, over their -OH groups converted from the enol-mold to the keto-mold, contribute an approachable hydrogen atom, which reduces the metallic ion into zero-valent NPs. Amongst those functional organic molecules, the phenolic groupings present in the extract more likely had an abundant impact on the metals [66], which reduced Ag^+^ to Ag^0^ [67]. The Fourier transform infrared (FTIR) of Parsley leaf extract (Figure 4b) comprises several clear peaks over the entire range. As a general rule, an FTIR spectrum has two regions, i.e., the functional group region (1800–4000 cm^−1^) along with the fingerprint region (0–1500 cm^−1^). The bands at 1645 cm^−1^ and 3383 cm^−1^ indicate C=O stretching of tertiary-amides and O-H stretching of phenol group, in that order [68]. A band at 1079 cm^−1^ indicates the CN stretching vibration of amines, whereas a distinct band at 1562 cm^−1^ specifies the bending of C-H bonds present in hydrocarbons [69] that follow the surface during Ag NPs production. The bands perceived at 1422 cm^−1^ and 667 cm^−1^ might be related to the C-H bending of alkanes and stretching vibrations of halo-alkanes, respectively [70].

### 3.2. Characterization of Ag NPs

In this investigation, several characterization techniques were used to study the structure, morphology, optical, thermal, and stability of Ag NPs.

#### 3.2.1. UV-Vis Spectrum of the Biosynthesized Ag NPs

**Figure 5:**
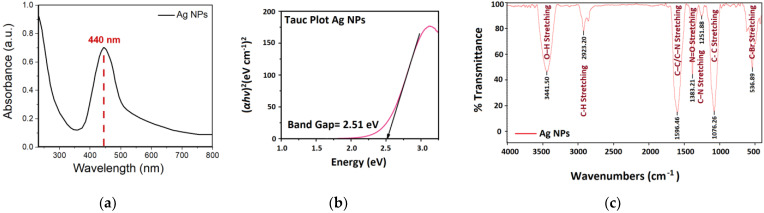
(**a**) UV-Vis spectrum, (**b**) corresponding Tauc plot for energy gap calculation, and (**c**) FTIR spectrum of biosynthesized Ag NPs using parsley extract.

Once the frequency of the electromagnetic field turns out to be resonant with the coherent electron motion, a robust absorption is created, which is the starting point of the observed color whose absorption extremely relying on particle size, dielectric medium, and the environments [71]. The UV-Vis absorption spectrum (Figure 5a) indicator of the creation of silver nanoparticles is at the visible range of 440 nm, while if we take the UV-Vis absorption spectra of silver nitrate, Ag (NO_3_), solutions, the absorption peaks will appear around 310 nm due to the existence of nitrate ions in the solution [72]. At the same time, in the Ag NPs case, both the conduction and valence bands would have lie down very nearby to each other, allowing the free flow of electrons that might have consequently given rise to the surface plasmon resonance (SPR) absorption band as demonstrated by Yallappa et al. [73], Ashraf et al. [74], and Devaraj et al. [75]. Moreover, the long tail, precisely at the long wavelengths, is a good indicator for having a tiny extent of aggregated particles, as reported by Hamedi et al. [76] and demonstrated in the investigation that the solution comprising NPs persisted unchanging for more than one month, with no indicator of aggregation or precipitation displaying the SPR peak at the equivalent wavelength [77]. Additionally, agglomeration is always the case, especially in the plant-mediated NPs synthesis, since the presence of biomolecules, particularly proteins, can easily modify the NP surface properties, leading to the loss of colloidal stability and formation of agglomerates. The absence of any additional peak in the UV-Vis spectrum is a good indicator of the purity of the biosynthesized Ag NPs. The results of this investigation agree with the previous studies, showing that the SPR peak for the spherical Ag NPs normally appears between 410 and 480 nm [72,78,79,80,81].

Furthermore, it can be seen from Figure 5a that the sharp peak is a clear indicator for the monodispersity of the biosynthesized Ag NPs [82]. The measured value is lower than that of bulk Ag particles, >100 nm, given as 520 nm and displaying a blue shift in excitonic-absorption, which specifies a tiny quantum confinement consequence [83].

Biological, green syntheses of NPs using plant extracts normally produces poly-disperse NPs with varied characteristics that are hard to regulate from an organic viewpoint. Therefore, the explanation of obtaining monodisperse Ag NPs using plant extracts is extremely significant. We have shown that the presence of polyphenols and flavonoid in parsley leaf extract affects the size and shape of Ag NPs. Polyphenols and flavonoid complex display superior reducing and capping properties than either polyphenols or flavonoid alone; therefore, this complex can produce homogenous, spherical, and monodisperse Ag NPs. In other words, parsley leaf extract was not arbitrarily selected in this study as a biosynthesizing medium for producing Ag NPs. Accordingly, in the first stage of the reaction, the polyphenols–flavonoid complex reduces silver ions to metallic silver, then the oxidized and none-oxidized forms of polyphenols–flavonoid complex stabilize the metallic nanocrystal and form stable Ag NPs.

The energy band gap of the biosynthesized Ag NPs was computed from Tauc’s plot by deducing the linear ratio of the UV-Vis curve. Figure 5b displays that the biosynthesized Ag NPs from parsley extract possess a value of energy band gap of 2.51 eV. These NPs with the relatively large value of band gap energy can be supplementary utilized in improved optoelectronic devices, thermal applications, batteries, and sensors as a semiconductor material. The corresponding band gap result is similar to the previously reported studies, and this value might be a result of the quantum confinement effect [84,85,86].

#### 3.2.2. FTIR Spectrum Analysis for Ag NPs

The FTIR spectrum of the biosynthesized Ag NPs is displayed in Figure 5c, which demonstrates absorption peaks positioned between the region around 4000 cm^−1^ and 500 cm^−1^. The FTIR spectra displayed absorption bands at 3441 cm^−1^, 2923 cm^−1^, 1596 cm^−1^, 1383 cm^−1^, 1251 cm^−1^, and 1076 cm^−1^, representing the existence of reducing, capping, and stabilizing biomolecules with the Ag NPs. The band at 3441 cm^−1^ in the spectra corresponds to the O-H stretching vibration, indicating the presence of alcohol and phenol [87]. The band at 2923 cm^−1^ was related to the C-H stretching vibrations of the primary and secondary amines [88]. The band at 1596 cm^−1^ is more likely related to C-C/C-N stretching vibrations of Alkene or amines [89], while the band at 1383 cm^−1^ is related to the N=O symmetry stretching typical of the nitro compound [90]. Additionally, the bands at 1251 cm^−1^ and 1076 cm^−1^ correspond to C-N and C-C stretching, indicating the presence of proteins amines [91]. Normally, the binding of protein with the Ag NPs maintains the stability of Ag NPs considerably through acting as capping and stabilizing agents and consequently protecting them from agglomeration [92]. Finally, the band at the 536 cm^−1^ region is most likely credited to C-Br stretching, which is typical of alkyl halides [93]. Other investigations reported that peaks at minor field in the range 400–700 cm^−1^ reflected the metallic nature of any examined sample, Ag NPs in our case [94]. As stated before, these functional groups, in general, have a role in the stability/capping of Ag NPs as reported in many studies [95,96,97,98].

#### 3.2.3. X-ray Diffraction (XRD) Pattern and X-ray Photoelectron Spectroscopy (XPS) of Ag NPs

**Figure 6:**
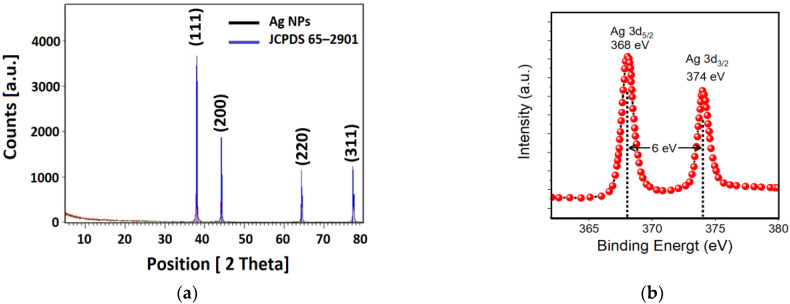
(**a**) X-ray diffraction patterns of Ag NPs and (**b**) XPS spectrum of Ag (0).

Figure 6a shows the X-ray diffraction (XRD) pattern of the biosynthesized Ag NPs. This pattern confirmed the crystallinity of the Ag NPs. The peaks at 38.01°, 44.34°, 65.52°, and 77.30° are equivalent to (*hkl*) planes of (111), (200), (220), and (311) planes, respectively. These peaks can be accredited to the face-centered cubic structure of silver nanocrystals, and they are in outstanding agreement with the previous studies [99,100]. It can be seen from Figure 6a that the diffraction pattern has been matched with JCPDS card No. 65–2901and all the diffraction peaks were indexed to a pure cubic Ag phase. In addition, there are no additional peaks observed in the XRD patterns indicating the high purity of the Ag NPs synthesized by parsley extract. This is a good indicator that plant extract, in contrary to other synthesizing methods, produces pure, high-quality, and stable NPs [13]. Furthermore, the purity of the sample has been proven by SEM and EDX analysis in the coming sections. The crystallite size and quality can be studied by investigating the full width at half maximum (FWHM) values from the XRD spectrum. Therefore, from the XRD spectrum, one can compute the average crystallite size using Debye–Scherer Equation (1) [101]:

(1) $$D=\frac{0.95\lambda }{\beta \;cos\theta }$$

where *D* is the average crystallite size, *λ* is the wavelength of the incident X-ray (0.154 nm), *θ* is the Bragg’s angle, and *β* is the full width at half maximum (FWHM). As a rule of thumb, the narrow FWHM, ~0.2475, is always a cue for having a high-quality structure of Ag NPs. The calculated average crystallite size of the Ag NPs around 30 nm. Comparable results have been found by other researchers [102,103]. The crystalline size is relatively smaller than the grain size, which can be obtained by the SEM. Thus, in order to calculate the actual nanoparticle size, TEM or DLS should be utilized [49]. XPS analysis was accomplished with the intention of studying the oxidation state of Ag NPs. The chemically reduced Ag designate peaks in the spectrum of XPS as displayed in Figure 6b at 368 and 374 eV, which can be allocated to Ag (0) 3d5/2 and Ag (0) 3d3/2, respectively [104]. This is another confirmation showing that Ag is in zero oxidation state, i.e., reduced silver, similarly confirmed by EDAX analysis in the coming section. It can be seen that from the XPS spectrum, nitrogen was not sensed from AgNO_3_, predictable at about 400 eV, demonstrating the creation of Ag NPs, and this outcome, again, was in good agreement with the EDX analysis.

#### 3.2.4. SEM and EDX Analysis of Ag NPs

**Figure 7:**
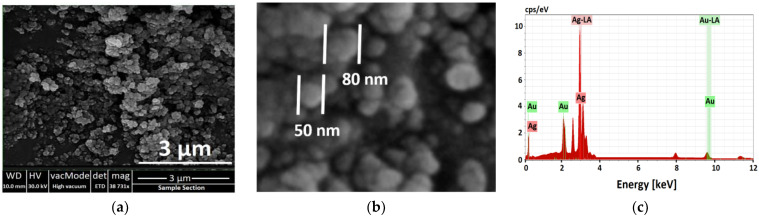
(**a**) Top view (**b**) higher magnification FE-SEM images and (**c**) EDX analysis of biosynthesized Ag NPs.

The morphology, shape, and size of the biosynthesized Ag NPs were investigated using a field emission scanning electron microscope (FE-SEM). It can be seen from Figure 7a that the synthesized Ag NPs possess uniformly dispersed spherical NPs. The grain size of the biosynthesized Ag NPs was in the range from 40 nm to 80 nm (Figure 7b); the real size of NPs can only be observed by dynamic light scattering (DLS) and transmission electron microscope (TEM) [105] since XRD provides the crystallite size, relatively smaller than the NPs size, and FE-SEM provides the grain size, comparatively bigger than the NPs size [49]. Figure 7a also shows some agglomeration states of the biosynthesized Ag NPs. Despite having a C-N and C-C stretching bond, as stated before, in the FT-IR spectrum, which is signifying the presence of proteins amines around the Ag NPs, they can protect the NPs from agglomeration, and there are still agglomerated clusters [92]. According to our best knowledge, as stated in the UV-Vis analysis, utilizing plant extract leads to a non-uniform nucleation process and thus forming the agglomerated clusters. On the other hand, agglomeration is usually owing to the high surface energy per unit volume ratio, which is the case in most biosynthesized NPs [6]. Our SEM results were in good agreement with those of preceding investigations [102,103].

Elemental analysis of the biosynthesized Ag NPs (Figure 7c) was investigated by Energy Dispersive X-ray (EDX) analysis. EDX spectra show a strong signal in the silver region, i.e., 3 keV, and confirm the formation of nanosilver and its elemental nature. This signal was shaped due to the excitation of surface plasmon resonance of Ag NPs. Some of the weak signals from Au were detected. These signals were formed owing to coating the Ag NPs with a 200 Å layer of gold to enhance the quality of the SEM images. Figure 7c shows the purity of the biosynthesized Ag NPs, which contains only an Ag element with no impurity from the other elements. Comparable analysis has been found by previous investigations [103].

#### 3.2.5. Transmission Electron Microscopy Analysis of Ag NPs

**Figure 8:**
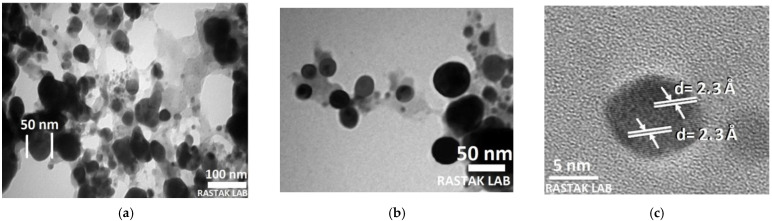
(**a**) HRTEM (**b**) closer observation HRTEM and (**c**) HRTEM image of individual Ag NPs.

To understand the impacts of the biosynthesis circumstances on the shape and size of Ag NPs, the morphology was investigated using transmission electron microscopy (TEM). Figure 8 displays the existence of spherical Ag NPs. TEM image shows an NPs size range from 40 to 60 nm. High-resolution TEM (HRTEM) study was utilized to find the structure of the biosynthesized Ag NPs. HRTEM displays the crystalline structure of single Ag NP, with noticeable lattice fringes. A lattice spacing of 2.3 Å was computed, equivalent to the Miller index plane (111) of face-centered cubic (FCC) Ag. The TEM images, similarly, revealed that the Ag NPs are monodispersed and highly crystalline, which is in good agreement with the SPR band in the UV-Vis and XRD spectra. It can be seen from Figure 8 that especially under close observation, the biosynthesized Ag NPs are surrounded by shaded layers of foreign matter, which most likely represent the capping agent from the parsley leaf extract. Additionally, the Ag NPs are separated from each other by a uniform interparticle distance. The results of HRTEM are in excellent agreement with an earlier investigation by Alahmad *et al.* [99].

#### 3.2.6. TGA–DSC Analysis

**Figure 9:**
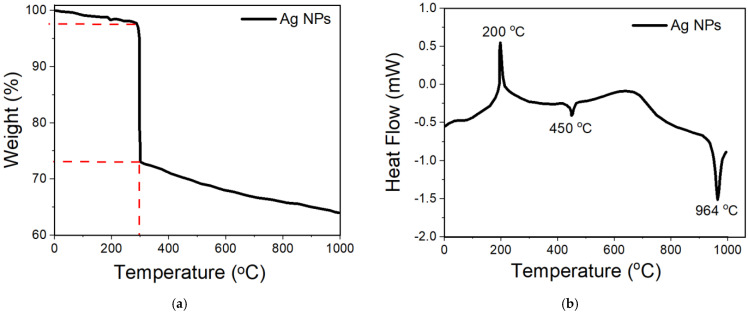
(**a**) TGA and (**b**) DSC curve of the biosynthesized Ag NPs.

It is well-known that in many applications, such as the current applications in this study, the thermal properties of nanoscale materials are extremely important. The impact of temperature in nanoscale materials has been studied widely, such as crystallization, melting, and decomposition points [106]. The purity and thermal stability of the biosynthesized Ag NPs were investigated using thermal gravimetric analysis (TGA) and differential scanning calorimetry (DSC) analysis (Figure 9). It can be noticed from Figure 9a that the first loss in mass of about 3% is related to the water desorption from the organic environment of the NPs, while the second loss in mass of about 27% demonstrated that the metallic core, Ag NPs, is enclosed by biomolecules. These outcomes agree in a high manner with the HRTEM results; it has been shown that the shaded layers are representing the capping agents surrounded around the Ag NPs. At the same time, these results solve the dilemma of stability of the available phytochemical inside the plant extracts, which are stable until high temperature [107]. In addition, TGA analysis (Figure 9a) proposed that the weight loss in the temperature range of 0 °C−100 °C is due to the water desorption from the organic environment of the NPs. This, in turn, proves that the biosynthesized Ag NPs are a good candidate for absorbing moisture in many areas of applications [108]. From Figure 9b, a sharp exothermic peak at 200 °C could be observed together with two small endothermic peaks, one at 450 °C and the other one at 964 °C (Figure 9b) analogous to the melting point of the metallic silver, which specifies the purity of the NPs. The deprivation pattern of organic compounds was lasting until 750 °C. In other words, there was no deprivation above 750 °C, which is related to the weight of Ag NPs. Analogous consequences have been found elsewhere by other researchers [109,110].

#### 3.2.7. Particle Size Distribution and Zeta Potential Measurement

**Figure 10:**
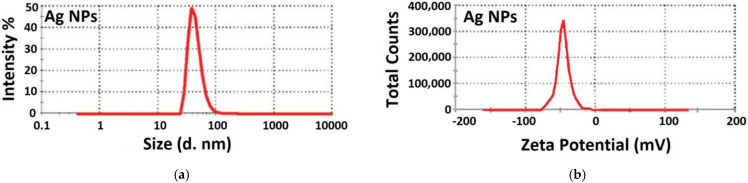
DLS analysis of the biosynthesized Ag NPs (**a**) size distribution (**b**) Zeta potential.

The dynamic light scattering (DLS) is utilized to compute the diameter of the Ag NPs dispersed in the liquid. The size dissemination profile of the biosynthesized silver nanoparticles measured by the DLS method is shown in Figure 10. The sharp signal confirms the equi-size particle distribution, an indicator for monodispersity, which agrees well with the UV–Vis spectrum. Size distribution profiles reveal one population of NPs with an average size around 55 nm. Zeta potential, in turn, is the degree of the actual electric charge around the surface of the NPs. Once a nanoparticle possesses a total surface charge, the charge is measured through the ion concentration within an opposite charge near the surface of the NPs [111]. The zeta potential value was shown to be −50 mV, which is corresponding to the high stability of the biosynthesized Ag NPs. These outcomes are in good agreement with Netala et al.’s results [112]. The DLS results coinciding with the range obtained from the HRTEM analyses. Consequently, the DLS and TEM analyses provided analogous results for the size range of the NPs. Parsley leaf extract mediated biosynthesized Ag NPs possess high negative zeta potential values, and thus, they are stable under a wide pH range [113].

### 3.3. Solar Energy Harvesting Using Ag NPs

Nanomaterials, in general, own better thermal, electrical, optical, magnetic, and mechanical properties, which have made them appropriate for countless applications [114]. For instance, in solar energy absorbers, nanostructured metallic materials are able, due to the high surface area per unit volume, to absorb the maximum incoming light flux through the vicinity of plasmonic structures [115]. In the surface plasmonic modes, the energy of the absorbed photons is given directly to the free electrons, and the produced hot electrons can be used in thermoelectric, photovoltaic, and photocatalytic platforms [116]. In other words, metallic nanoparticles can enhance the absorption and emission of light and then provide local heating.

**Figure 11:**
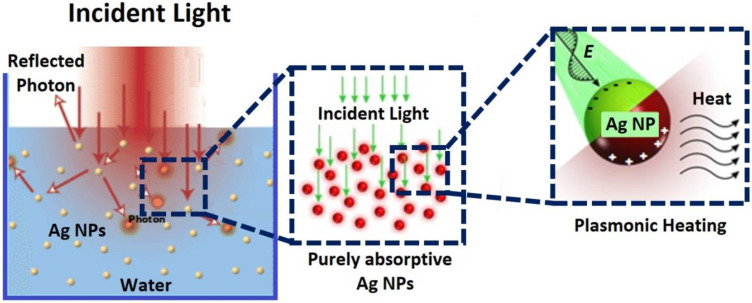
Schematic representation of heat generation from the Ag NPs in water when exposed to the sunlight.

The plasmonic photothermal characteristics of metallic NPs are of massive attention in biomedical areas since of their robust optical reaction and the aptitude to operate the photothermal consequence through the exterior light sources [117]. In addition, the most important role of plasmonic effects in hot electron production lies in their aptitude to harvest near-infrared and infrared regions of the solar spectrum, which cannot be obtained in conventional photocatalytic devices [118]. Among the metallic nanostructured materials, NPs have played, exclusively, an important role in the development of photothermal generation devices. Nanoparticles such as gold and silver can effectively release heat under the light source [119]. The heat generation is due to the plasmonic electric field driving the electrons forcefully inside the nanocrystals, while the light energy received by the Ag NPs is converted to heat through the interaction between light and the mobile charge carriers in metallic nanoparticles and increase the temperature of the surrounding medium as shown in Figure 11 [120].

The reason behind selecting Ag NPs for this purpose is that Ag NPs, after Au NPs, are the best candidate for the plasmonic heating generation process [117]. Meanwhile, in semiconductor NPs, the heat release rate is much weaker since heat is produced over the interband absorption procedure with the formation of mobile electrons and holes [121].

As stated previously, the utilized biosynthesized method in this study is producing spherical Ag NPs. Thus, the movement of electrons in the electric field results in the polarization of the sphere; therefore, there is a linear restoring force, which reaches the extreme point at the SPR. Accordingly, in contrast to the bulk material, a free electron in the spherical particles is a vibrational system [122,123]. 

**Figure 12:**
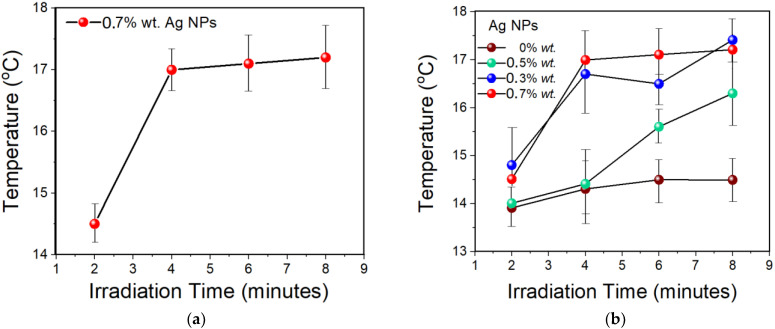
(**a**) Temperature profiles of 0.7% *wt.* Ag NPs; (**b**) different percentages of Ag NPs in water medium under sunlight.

Figure 12a shows temperature profiles of 0.7% *wt.* Ag NPs in water medium by heat generation due to SPR of free electrons when exposed to the sunlight. It can be noticed that the heat released by the Ag nanofluid under sunlight irradiation at the plasmonic-resonant wavelength increased the temperature of the water medium as the irradiation time increases [124]. In order to achieve an optimal plasmonic heating generation, the wavelength of the light source should be close to the plasmonic resonance wavelength of Ag NPs [125]. It can be seen from Figure 12b that the released heat by the Ag nanofluid under sunlight irradiation at the plasmonic-resonant wavelength increased the temperature of the water medium as the irradiation time increased. Additionally, the concentration of Ag NPs has a noticeable effect on increasing the water temperature. For instance, at a low concentration of Ag NPs (0.3%), the temperature of the water rises up to 17.4 °C after 8 min, while the temperature profiles containing between 0.5% and 0.7% of Ag NPs were reached at 16.3 and 17.2 °C, respectively. As a result of the absorption, trapping, and scattering of the incident light by the Ag NPs over a board spectrum is following the generation of heat through non-radiative damping after sunlight absorption, as explained in Figure 11 [124].

Beicker et al. studied the photothermal conversion behavior of gold/water and also multi-walled carbon nanotube (MWCNT)/water nanofluids at specific volumetric concentrations (0.0001–0.004% and 0.0001–0.03%, individually [126]. They showed that the optimal nanoparticle volumetric concentration was 0.002% for the gold nanofluids and 0.001% for the MWCNT. These results indicate that by optimization, the solar spectrum could be absorbed to its maximum level. However, He et al. used Cu nanofluid as solar absorber nanoparticles, and they showed the efficiency of solar collectors in the water medium was improved to 23.83% for (25 nm, 0.1 wt. %) Cu NPs [127]. Moreover, they also showed that the optical absorption of metallic NPs could be improved more by merging with graphene. Recently, different research groups synthesized nanofluids based on spherical silver, gold, and copper NPs, covered by graphene oxides (GO) structures and studied their thermal absorption behavior under the sunlight [128]. These results demonstrated that the new nanostructured materials could convert solar energy to thermal energy under low and high solar irradiation. As stated by Campos et al. [128], the nanofluids were increased their temperature and reaching the boiling point after 10 min. In fact, this is already expected by nanomaterials due to their extraordinary physical properties, such as large surface area per volume. In addition, when the scale of the nanoparticles is comparable to the free electrons De-Broglie wavelength, the periodic boundary conditions would be changed due to the size shrinking. Thus, light absorption, magnetic effect, thermal conductivity, chemical reactivity, and melting point can be converting strongly. For instance, the properties of plasmonic resonance frequency change with different particle sizes; therefore, the NPs size can be tuned to control photothermal conversion with broadband sunlight absorption [129].

## 4. Conclusions

In this study, high purity, thermally stable, monodisperse, and spherical Ag NPs were synthesized from a simple, rapid, safe, and one-pot green method using parsley leaf extract. Different characterization techniques were utilized to investigate the morphology, purity, stability, crystal structure, optical, and thermal properties of the biosynthesized Ag NPs. This investigation also shows that polyphenols and flavonoid complex exhibit higher reducing and capping properties than either polyphenols or flavonoid alone; thus, this complex can provide homogenous, spherical, and monodisperse silver NPs. The biosynthesized Ag NPs were used to investigate the plasmonic effect of different concentrations, namely 0%, 0.3%, 0.5%, and 0.7% Ag NPs and monitor the released heat from nanoparticles into water medium through irradiation under the sunlight. It was found that the temperature of water ambient reached 17.4 °C when 0.3% of Ag NPs were used within 8 min of irradiation. In addition, this study showed that the released heat by the biosynthesized Ag NPs from the irradiation around 450 nm leads to the thermal decomposition of localized surface plasmon resonance. Finally, the result of this investigation is promising, and it emphasizes that the Ag NPs can be utilized as converting sources of solar energy to thermal energy and then increasing the temperature of the water medium at optimized conditions.

## Figures and Tables

**Table 1:** Temperature measurements profile of water using different concentrations of Ag NPs.

Water mL	Ag NPs Concentration	Temp. (2 min) °C	Temp. (4 min) °C	Temp. (6 min) °C	Temp. (8 min) °C
50	0.0%	14	14.3	14.5	14.8
50	0.3%	14.8	16.7	16.5	17.4
50	0.5%	14	14.4	15.6	16.3
50	0.7%	14.5	17	17.1	17.2

## Data Availability

This work is part of an MSc Project; thus, the data are kept.
